# Public Health Ethics and ‘the Science’ in Public Decision-Making

**DOI:** 10.1093/eurpub/ckac129.129

**Published:** 2022-10-25

**Authors:** J Coggon

**Affiliations:** 1 Centre for Health, Law, and Society, University of Bristol Law School, Bristol, UK; 2 UK Faculty of Public Health, London, UK

## Abstract

In this presentation, I aim to complement the ideas presented by the other speakers by raising questions about ethics and (different senses of) integrity. In particular, against the pressures of providing evidence bases for governmental decision-making during the COVID-19 pandemic, I ask critically whether, or to what extent, we find harmony between the integrity of science, scientists, and democratic decision-makers. The potential for tensions is explained through consideration of public communication, pluralism in scientific knowledge bases, basic uncertainty, and fundamental principles such as transparency in political decisions. The reality of the tensions is found by considering some limits of ‘the science’ as it has been used in political turns of phrases such as ‘we are following the science’. The tensions are particularly evident where there are gaps given expedited methodologies, when we explore how ‘the science’ actually represents a plural concept, and where we acknowledge that science even broadly conceived is not able to be all that leads decision-making. And they are compounded by factors that might motivate silence or simplification in public communication, such as the apparent appeal of making ‘the science’ seem clearer or more uniformly agreed than may be the case, or by using ‘the science’ to avoid or obfuscate discussion of value judgments. The complexities of these ethical points provide their own important context for evaluating evidence-based policy within and beyond the pandemic.

